# Using the South African Triage Scale for prehospital triage: a qualitative study

**DOI:** 10.1186/s12873-021-00522-3

**Published:** 2021-10-30

**Authors:** Julia Dixon, Taylor Burkholder, Jennifer Pigoga, Michael Lee, Kubendhren Moodley, Shaheem de Vries, Lee Wallis, Nee-Kofi Mould-Millman

**Affiliations:** 1 School of Medicine, Department of Emergency Medicine, University of Colorado Denver, 12631 E 17th Ave, Room 2612, MS C326, Aurora, CO 80045 USA; 2 Keck School of Medicine, Department of Emergency Medicine, University of Southern California, California, Los Angeles USA; 3 Department of Surgery, Division of Emergency Medicine, University of Cape Town, Cape Town, South Africa; 4 Department of Health, Emergency Medical Services, Western Cape Government, Cape Town, South Africa

**Keywords:** Prehospital, Triage, EMS, SATS; South African Triage Scale, Focus groups

## Abstract

**Background:**

Triage is a critical component of prehospital emergency care. Effective triage of patients allows them to receive appropriate care and to judiciously use personnel and hospital resources. In many low-resource settings prehospital triage serves an additional role of determining the level of destination facility. In South Africa, the Western Cape Government innovatively implemented the South African Triage Scale (SATS) in the public Emergency Medical Services (EMS) service in 2012. The prehospital provider perspectives and experiences of using SATS in the field have not been previously studied.

**Methods:**

In this qualitative study, focus group discussions with cohorts of basic, intermediate and advanced life support prehospital providers were conducted and transcribed. A content analysis using an inductive approach was used to code transcripts and identify themes.

**Results:**

15 EMS providers participated in three focus group discussions. Data saturation was reached and four major themes emerged from the qualitative analysis: Implementation and use of SATS; Effectiveness of SATS; Limitations of the discriminator; and Special EMS considerations. Participants overall felt that SATS was easy to use and allowed improved communication with hospital providers during patient handover. Participants, however, described many clinical cases when their clinical gestalt triaged the patient to a different clinical acuity than generated by SATS. Additionally, they stated many clinical discriminators were too subjective to effectively apply or covered too broad a range of clinical severity (e.g., ingestions). Participants provided examples of how the prehospital environment presents additional challenges to using SATS such as changing patient clinical conditions, transport times and social needs of patients.

**Conclusions:**

Overall, participants felt that SATS was an effective tool in prehospital emergency care. However, they described many clinical scenarios where SATS was in conflict with their own assessment, the clinical care needs of the patient or the available prehospital and hospital resources. Many of the identified challenges to using SATS in the prehospital environment could be improved with small changes to SATS and provider re-training.

**Supplementary Information:**

The online version contains supplementary material available at 10.1186/s12873-021-00522-3.

## Background

**Fig. 1:**
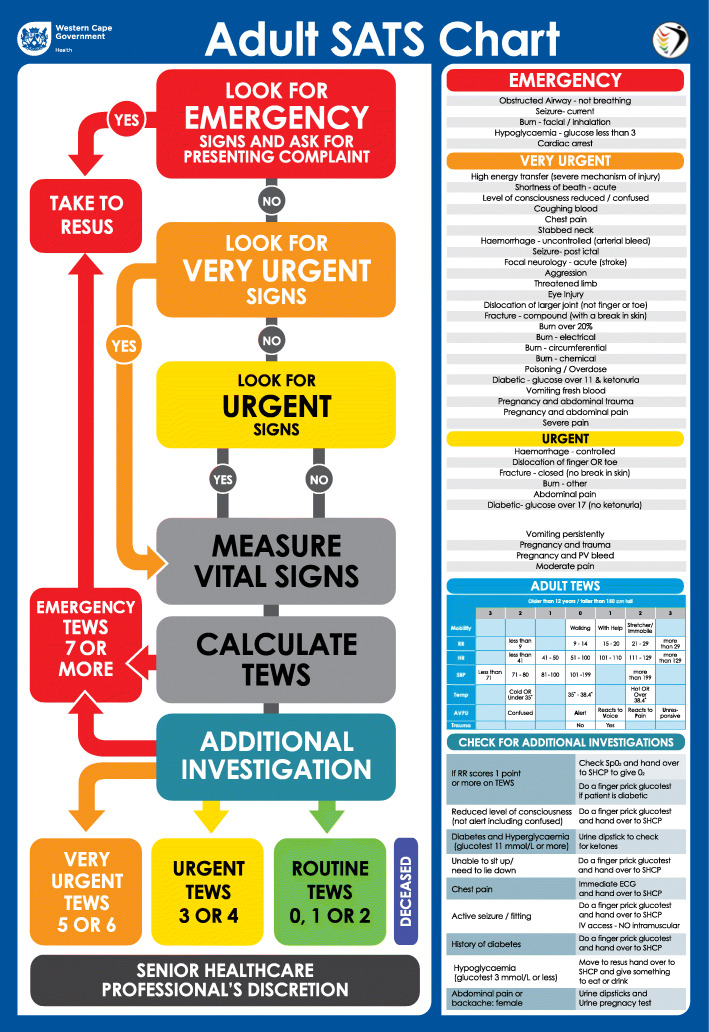
South Africa Triage Scale chart for adult patients [1]

The South African Triage Scale (SATS) was developed to triage undifferentiated acute care patients presenting to facilities in low-resourced African settings [1]. SATS was created for, and first validated amongst, in-hospital emergency care providers (physicians and nurses) in South Africa [2–6]. To determine the final SATS triage acuity, a Triage Early Warning Score (TEWS), including variables mobility, heart rate, respiratory rate, systolic blood pressure, temperature, mental status and presence of trauma is calculated. Each score is associated with a SATS colour, green, yellow, orange, red from lowest to highest acuity respectively and blue used for patients without signs of life. Additionally, the SATS colour can be upgraded to a higher acuity using a list of 32 high risk clinical discriminators (Fig. 1) [1]. In validation studies, SATS had an over triage rate of 15% and under triage rate of 10% when tested among South African in-hospital emergency centre providers [2, 3].

The Western Cape Government (WCG) Emergency Medical Services (EMS) system is a government-operated EMS system responsible for providing ambulance services in Western Cape Province of South Africa. In 2017, WCG EMS executed approximately 450,000 ambulance responses, which includes primary responses (i.e. to the scene) and inter-hospital transfers [7]. WCG EMS is staffed by basic life support (BLS), intermediate life support (ILS) and advanced life support (ALS) providers, the scope of practice for these providers is consistent with other international EMS services. In 2012, SATS was formally and innovatively incorporated into prehospital emergency care by the WCG EMS system as a tool to guide both patient triage and destination decision making [8]. Lower acuity (SATS green and yellow) patients are usually triaged to district hospitals and higher acuity patients (SATS orange and red) are triaged to regional and tertiary facilities. Although prehospital providers have the resources and skills to derive the SATS triage acuity, it was never intended for use in, nor formally adapted for, prehospital care. Prior work with WCG EMS that was not designed to evaluate pre-hospital triage as a primary outcome suggested that SATS has limitations and challenges when used in a pre-hospital setting, particularly related to use of the discriminator [9].

In the prehospital setting, ideally an effective triage instrument can objectively and accurately determine clinical acuity, standardize handover communication, and guide the selection of an appropriate destination facility [10]. Consequences of incorrect application of a triage tool include under-triage (i.e., under-estimating true acuity), which can be harmful to patients or over-triage (i.e., over-estimating true acuity), which may result in wasted resources [10–12]. The only prior study of prehospital SATS, conducted in WCG EMS, reported an acceptable (13%) over-triage rate and a high (30%) under-triage rate, the latter largely attributed to misuse of the clinical discriminators [8]. However, the providers’ perspectives and personal experiences using SATS as a prehospital triage tool remain unstudied.

A strong and practical need exists to assess the prehospital provider perspectives and experience of using SATS in the WCG EMS system in order to identify potential improvements in pre-hospital triage. Findings from this study may also have application and implications to prehospital systems in other resource-limited settings. Tiered health systems, common in low-resource settings have a dual need for triage tools to both assess patient acuity and determine a destination facility. An effective pre-hospital tool can help facilitate the provision of high-quality emergency care and optimize utilization of limited health resources [10].

### Objective

We sought to qualitatively describe EMS providers’ perspectives and experiences of using SATS as a prehospital triage tool in the Western Cape. Conducting focus groups and applying content analysis methodology allows for a more in depth understanding of how pre-hospital providers use SATS, situations in which use of SATS may be challenging and problematic, and how pre-hospital providers feel that it could be improved.

## Methods

Focus group discussions with WCG EMS providers were conducted in March and April, 2017. Participants for the focus groups were deliberately cohorted according to the three training tiers; BLS, ILS and ALS, to allow participants to share and discuss perspectives that may be unique to their level of training and to prevent perceived hierarchy from limiting participation of less experienced providers. Focus group participants were recruited from a convenience sample of WCG EMS personnel attending continuing education courses at the WCG EMS College of Emergency Care. None of the focus group participants were actively enrolled in courses at the College of Emergency Care that involved triage nor use of SATS [Personal communication, Michele Kings, Western Cape College of Emergency Care].

An interview guide was developed by the study investigators, and interview questions were confidentially reviewed and revised by a small group of WCG EMS staff (not participating in the study) to ensure focus group questions were phrased in a contextually, grammatically and culturally appropriate fashion. The interview guide was organized according to questions with similar topics e.g., initial SATS training, components of SATS, challenges and facilitators of using SATS in the field and suggestions for improvement. (Appendix) The focus group discussions were conducted in a private room at the College of Emergency Care and led jointly by a US study investigator (JD and NM) and a trained WCG EMS staff member (ML). Focus group discussions were designed to last approximately 90 min and were conducted in English, and terms in other languages (Afrikaans, Xhosa) were clarified by the WCG EMS staff member in real-time. Participants were provided with the SATS poster which serves as visual aid summarizing the SATS process and routinely available on WCS EMS ambulances. All focus group sessions were audio recorded, and supplementary field notes were taken by the interviewer. Focus groups were transcribed verbatim in English by a study investigator into Microsoft Word and verified for accuracy by a second investigator.

### Qualitative analysis

Content analysis with an inductive approach was selected due to investigators previous exposure to, and observations of, prehospital SATS used by WCG EMS providers. Three study investigators (JD, TB, NM) developed a code book using content analysis methodology [13]. In conventional content analysis the categories and names of categories are derived from the data. Main themes, also called categories, can contain several sub themes that further describe components of the main theme [13]. Coded transcripts were entered into Atlas. Ti (ATLAS.ti Scientific Software Development GmbH, Version 8.4.4) and analyzed for themes and categories within themes as they related to the primary research question of the EMS experience of using SATS. Each transcript was iteratively coded by at least two reviewers independently (JD, TB, NM) who each came up with their own descriptive codes. All three team members met to discuss identified codes and develop consensus definitions of codes. The refined codes were then applied to each manuscript. Subsequently, three reviewers discussed codes, identified themes and sub-themes. Differences were resolved by study team consensus.

### Ethical approvals

Full research and ethical clearance was obtained from the University of Cape Town (HREC# 705/2015) and Colorado Multiple Institutional Review Board (COMIRB Protocol 16–2271). Written institutional approval was also obtained from WCG EMS. Written informed consent was obtained from all participants.

## Results

A total of three focus groups were conducted, each consisting of five WCG EMS providers. All participants had prior experience using SATS in the prehospital setting. 11 participants were male; 8 participants were from rural and 7 from urban EMS bases (ILS group: 4 male 1 female, 3 rural, 2 urban; BLS group: 2 female, 3 male, 4 rural and 1 urban; ALS group: 4 male 1 female, 4 urban, 1 rural). Thematic saturation was reached within these 3 groups. Prehospital providers’ perspectives and experiences of using SATS emerged according to the following themes: implementation of SATS, perceived effectiveness of SATS, use and limitations of the clinical discriminator, and special EMS considerations. (Table 1).

**Table 1:** Identified themes and sub-themes

Theme	Implementation of SATS	Perceived Effectiveness of SATS	Use and limitations of the clinical discriminator	Special EMS considerations
Sub Themes	n/a	Situations when SATS works well	Experiences using the discriminator	Dynamic clinical care in the field
		Situations when SATS does not work	Challenging discriminators	Communication with facilities
			Field vs Hospital acuity	Socio-cultural factors in conflict with SATS

### Implementation and use of SATS

Several participants described either formal classroom training on SATS while others described no formal training at all and learned how to use the tool from their peers or on their own. Participants stated they often refer to the SATS reference card, or poster on the ambulance wall, to help determine the triage acuity of their patients. Participants from all focus groups described how SATS is used as both a triage and destination decision making tool: the triage acuity often helps to determine the level of destination facility. Participants noted that, specifically for trauma, there are WCG protocols that determine destination facility instead of SATS, however many also noted that they rarely use the trauma protocol and instead use SATS.

BLS E: I actually had a [*continuing medical education*] workshops where all of the people we had to go sit in a classroom for an hour or day, and they gave us a little book where you, it actually explains the entire process of TEWS and SATS.

ILS E: It was nothing formal. Just basically learning it on the ambulance. Well, TEWS we were explained. But SATS worked was actually filling it out on our report forms. So it was more learn by doing.

ALS C: Yes, because you can … like, if it’s a green patient, you taken them to a day hospital. Yellow up, you take them to the appropriate facilities. So, triage makes it, makes our job much easier when it comes to sorting out the patients.

ILS B: Also there is a lot of information to remember so I always defer, I have one on my clip board that I refer back.

### Perceived effectiveness of SATS

#### When SATS works well

Overall, participants stated that SATS was a useful prehospital triage tool that performed positively in a large portion of cases. Participants reported that TEWS was the easiest part of the tool to use because it utilized objective vital sign data. Respondents explained that SATS provides them with a consistent language and objective tool that promotes communication and patient handover with facility providers. Participants also felt that SATS helped identify patients who initially appeared ‘not sick’ but may have abnormal vital signs – this situation results in providers revising their initial gestalt to recognize patients that require higher triage acuity.

ALS C: It’s actually nice, the TEWS … your patient is actually green, but now has got severe abdominal pain, which makes him now … you upgrade him to a yellow. Which means he doesn’t go to a day hospital now, but he goes to a tertiary hospital. When the doctor say, “Why are you bringing this patient here?,” you say “According to the discriminator, he’s yellow/orange … he’s supposed to be here.”

BLS D: I mean especially your patients that seem green especially when you arrive on scene and they are walking and talking, let’s say an abdominal complaint or something like that, and sometimes when you actually sort of go through TEWS you pick up with a heart rate or respiratory rate or something like that, that they are actually to be triaged more toward yellow or orange, sometimes even red. Those are the times when I really have felt it’s made an effect on how I change my sheet.

ILS D: When it comes to give over, for the doctors, for them accepting or not accepting a patient. I think it helps.

ALS B: Very rarely do you find it’s like 100% completely off kilter. It’s more than, like, close to what you need or the patient needs in the end.

#### When SATS does not work

Participants also described patient scenarios when SATS was not a helpful tool, particularly for the care of trauma patients. Multiple participants gave examples of trauma patients with a SATS triage of green or yellow but who were clearly going to need surgical care at a higher level of facility. In both trauma and medical cases, when the provider gestalt of the patient’s acuity did not match the SATS final triage acuity, participants in all focus groups described how they adjusted the TEWS score by one or two points, such as marking the patient as not mobile because they were on the stretcher, to justify taking the patients to the facility they felt was most appropriate. Several participants also stated that SATS was limited because it did not represent an accurate clinical picture of the patient in front of them which they felt was the most important factor for triage and destination decision making.

BLS D: I think TEWS personally, is more for my medical patients. I use trauma patients, I basically, I triage them by myself as they present and how I see it. After I’ve done my own assessment and things like that, because, once again, it’s a piece of paper, it can’t say what the mechanism of injury was.

ALS D: The thing is, it’s also the alertness as well. The AVPU can also be a little subjective at times.

ILS E: I have had red patients on TEWS and they looked perfect. I’ve had patients that are yellow to green on TEWS and they need a hospital immediately. So the thing with a red patient to me, is that is my interpretation of it. How important is it to have this patient in the hospital? Is there anything I can do?

### Experiences with the SATS discriminator

#### Using the discriminator

Participants had the broadest range of experiences when it came to using the clinical discriminators. Some reported they rarely used discriminators while others described always writing in some type of discriminator, even if none of the SATS discriminators applied to the clinical scenario they wished to describe. The self-created discriminator was discussed in focus groups with all levels of providers. Participants explained that they often used a novel free text discriminator to help justify their choice of destination facility. For rural participants, this was important when they could identify a patient’s resource need (e.g., x-ray) and match the patient’s need to the resource-available facility in their community, regardless of the SATS acuity.

ILS E: I will say if you’ve got a round hole and you try to plug it, [SATS] is a square peg. It fits it. But it’s not right. I can understand somehow in a hospital setting it works really well. You’ve got various departments and those discriminators are at the discretion of a doctor or the physician attending. But we don’t have that, that discretion. So we are given these discriminators to kind of fill in.

BLS B: As the TEWS was scoring green or a yellow, we going to score maybe a red because we see this is maybe a life-threatening thing. That is how we discriminate. This is life-threatening and then we see the patient need the help as soon as possible, so then we discriminate to the red.

BLS D: I’ve noticed that sometimes you don’t get what you’re looking for in the discriminators. You put in your own discriminator because you feel, once again, the patient doesn’t present what, the patient might triage green, you actually can see by the presentation, because I mean, this is a piece of paper, it can’t see the patient.

#### Challenging discriminators

Participants stated one of the most subjective and difficult components of SATS was the discriminator. One reason given by participants for difficulty in using the discriminator is unavailability of point-of-care testing, which is used for in-hospital triage, such as ketones in the urine, and rapid pregnancy tests. Hence, they cannot apply discriminators appropriately in the prehospital setting. Participants also explained that other discriminators are often very subjective and hard to apply to patients consistently; examples include ‘severe pain’, ‘chest pain’ and ‘abdominal pain’. Participants also described how the trauma discriminators (e.g., ‘high energy transfer’ and ‘fracture - closed’) were difficult to use due to their highly subjective interpretation and for fear that trauma patients can rapidly decline during transport.

ALS B: Very rarely will people use severe pain, using it as a discriminator for taking a patient to a particular facility. I don’t think. I think people, either they’ve used it and it hasn’t worked for them because the doctors don’t like it or they just don’t consider it as a discriminator.

ILS B: But there is also times when there are ambulances that are not enough … There is diabetic glucose of 11. What do you do? We don’t have a test like that for the urine.

ILS C: There are some calls that don’t have applications and what you fill in and guess because the discriminator is a major player factor with regards to your TEWS. Because I find just look at the presentation of the patient and the patient does not look well.

#### Field versus hospital acuity

Participants also mentioned that the acuity level associated with discriminators did not seem to be a good fit for the acuity of certain clinical conditions in the prehospital environment. For example, ‘seizures-post ictal’, in the hospital yields an orange SATS final triage score, however, in the back of the ambulance, the limited clinical scope of providers and chances of the patient having another seizure led some prehospital providers to believe these patients should be classified with higher acuity (e.g., red). Participants mentioned that other clinical discriminators which proved difficult to use are burns and ingestions due to the broad range of clinical presentations and severity associated with each. Participants explained that discriminators should allow providers to differentiate within a discriminator category, severe cases from less severe cases based on their clinical assessment.

ALS C: I’ve never used burns, I guess you know, you end up doing vital signs on them anyways and they tend to score in the 7’s [for TEWS] anyways a lot of the times I’ve had them, you know. So, I’ve never really had to use discriminator. I never find myself needing to look at the list with those sorts of patients because it’s usually quite obvious that they’re critical we’ve got to go somewhere.

BLS E: I think most of the times it’s helpful in our situation to upgrade the colour, because there is only so much you can do for a patient in a certain situation, so if they are scored higher, they can either upgrade our vehicle to the paramedic vehicle or to a higher qualification vehicle, or the hospital knows what’s coming, and they can prepare.

BLS D: Or the whole score, I think should be more humanized. You know, because now it’s paper. For me it feels like a piece of paper that tells you this is where patient should be. Where I feel that any score should be more human and that actual piece of paper is. Nobody presents the same.

### Special EMS considerations

Participants identified challenges of using SATS due to the unique prehospital environment. These challenges were often nuanced and due to a combination of the providers’ expertise, resources available, patient circumstances and the relationship with the facility. BLS, ILS and ALS providers have different scopes of practice and all provide care with a limited amount of resources that impacts their field decision making. Providers from all cohorts described using SATS in an adjusted way that justified and facilitated faster access to a facility or additional resources when needed.

#### Dynamic clinical care in the field

Participants also gave multiple examples of decompensating patients in the field complicated by long transport times and challenged by limited in-ambulance resources. Participants reported an awareness of how rapidly a patient’s condition can change and that providing care is more difficult in the back of an ambulance compared to within a facility. Additionally, participants described a lack of understanding by the facility personnel about their EMS scope of practice, often resulting in facility personnel questioning why an intervention was not delivered or why the patient was brought to their facility and not another.

BLS E: You can control bleeding or whatever, but trauma is a continuous thing. You can fix one thing and something else can go wrong. So it’s a continuous process.

ILS C: I think the other thing is you are sitting with two environments. One is the unstable environment where we are working so you take this red patient, according to you. You come in and take the patient to a doctor, which is highly qualified and a sister. They look at you and say “Why is the patient red? they are stable.” So the same red for me is not the same red for them. I think that is a big difference between hospital and myself. The stability of the environment.

ALS A: The expectation is that we are supposed to diagnoses the patient and that’s the also a little unreasonable cause we’re not supposed to diagnose. We’re not qualified to diagnoses people. It’s not our job. We triage them. That’s essentially what we do. We go and triage and take them somewhere. That’s it!

#### Communication with facilities

Participants described a broad range of experiences with SATS when communicating with receiving facilities. Participants stated that the hospital staff often challenged their SATS assessment and some reported the hospital re-calculated the triage acuity and sent them to another facility. Others reported a more positive relationship with the facilities and facility providers’ willingness to collaborate and help the patient. The experience of using SATS to determine the destination facility was different for rural and urban providers. Several rural participants described being told by one level of facility to take the patient to a different level of facility that can be more than an hour away.

ILS D: Problem is that he’s stable now, but after the 1.5h drive to the hospital, one will not take me. Another 1.5h away from the hospital for a secondary call. Then the patient is going to be three hours in the ambulance when they could have spent half their time in the hospital already.

ALS B: But you know, and then you go face the barrage of attacks but it’s not just all about SATS, it’s about the patient sometimes.

BLS B: It seems like they don’t know, they don’t actually have an idea of how do I do it on the road. They just sometimes think of us as drivers. They don’t have a better idea of what we actually do to the patient.

#### Socio-cultural factors in conflict with SATS

Participants described many scenarios in which the socio-cultural patient factors were in direct conflict with the SATS driven destination. Multiple participants described examples of patients from rural communities or with chronic illness who either refused to go to a high level facility due to anticipated difficulty with return transportation or who could benefit from care at a higher level facility despite being scored as green or yellow.

ALS B: You know it can be kind of lame if you spend all of your time following a chart to make a decision. And having the freedom to go, “Ok, maybe this patient could use something different and maybe there is space to decide. Maybe it’s more inclined to be a yellow-type abdominal pain kind of thing even though abdominal pain is always yellow.’ Someone suffering from abdominal pain for the last six months and no one has helped them with it, maybe it’s time they go somewhere a little better than the CHC to get the help they need.

ILS D: When I am working in a big town and there are small towns around there with clinics. Then you pick up the patient on a farm. But then you tell the patient you need to go to the hospital, they tell you they don’t have the money to go there.

## Discussion

To our knowledge, this study is the first to investigate the perspectives and experiences of prehospital providers using SATS in the out-of-hospital setting. The study findings advance our understanding of the adoption of SATS into a prehospital clinical environment for which it was not initially designed. Overall, participants spoke positively about many aspects of their experience with SATS, but they also recognized issues regarding the EMS implementation, clinical application and health systems consequences of using SATS in the prehospital setting. Many of the prehospital challenges with SATS are related to its use as a decision tool for destination facility selection which also determines what resources are available to the patient once they arrive at the facility.

The identified thematic areas — implementation and use of SATS, perceived effectiveness of SATS, use and limitations of the clinical discriminator, and special EMS considerations — are critical to understanding how SATS is actually used in the field and what components of SATS require modification for the prehospital setting.

### Implementation and use of SATS

SATS was implemented into WCG EMS with only brief formal training for those employed by the agency at the time of implementation and largely informal peer-to-peer training on the job for those who subsequently joined. A previous study of 102 WCG EMS providers found an unacceptable under-triage rate of 29.5%, much higher than similar studies of doctors and nurses in-hospital [8]. While EMS provider qualification levels and differences in scope of practice may contribute to the poorer prehospital SATS performance, we posit that on-going formal training, including SATS competency assessments, are important for optimal use of SATS by prehospital providers in the long-term. Additionally, we posit the challenges identified as other themes in this study reflect EMS providers reliance on their gestalt, facility considerations, and socio-cultural factors considered during assignment of a triage colour and that re-training alone would not improve performance. It is important to note that previous in-hospital validation studies of SATS relied on doctors, nurses, and nursing assistants who had undergone formal training sessions prior to validation assessments [3, 5, 6].

Participants practicing in urban areas with many potential destination facilities discussed the beneficial use of SATS to help select the most appropriate destination. Inappropriate selection of the destination facility has been found to be a major issue in other prehospital systems in Africa [14]. Participants indicated that being able to use the tool to select an appropriate destination was valuable, although issues arose with certain types of patients (e.g., traumatic injuries). SATS did not help providers advocate for their choice of destination when facilities declined to accept the patient—either due to a mismatch of understanding of which SATS levels were appropriate to transfer to their facility or to a re-calculation of SATS upon arrival to the facility. The latter could be addressed by issuing or clarifying guidance to WCG EMS providers and hospital personnel as the SATS-based destination decision-making. Issues with trauma patients are already addressed through a WCG EMS protocol for transporting injured patients. The integration of SATS triage acuity with other protocols may help prehospital providers determine best destinations and better optimize health system resource utilization, with unique considerations for rural settings.

### Perceived effectiveness of SATS

Most providers described being able to use SATS effectively and felt SATS helps them identify high-acuity patients and communicate in shared terminology with the hospital providers who also use SATS for triage. In contrast to this perceived ease of TEWS, it was recently noted that it is often under-calculated by WCG EMS personnel, particularly for trauma and high-acuity patients [8]. We did not identify a potential reason for this in the focus groups, although simple computational errors while multi-tasking under stress have been proposed as one potential cause. Additional clinical studies may be warranted to better understand this, and an audit and feedback program may potentially help improve accuracy.

### Use and limitations of the SATS discriminator

The list of clinical discriminators was originally developed for use by frontline in-hospital clinicians (doctors and nurses). Clinical discriminators are intended to identify high-risk clinical conditions that are otherwise not captured by TEWS and require higher medical priority or more resources [15]. During previous validation of the use of SATS by WCG EMS providers, it was found that clinical discriminators were often missing or incorrectly applied. Several trauma-related discriminators (e.g., ‘high energy transfer‘, ‘burn circumferential‘and ‘haemorrhage controlled‘) were among the least frequent to be correctly applied [8]. In our study, participants echoed difficulties with applying the discriminators due to subjectivity (e.g. ‘severe pain’) and the requirement of advanced diagnostic studies (e.g., ‘diabetic – glucose over 17 (no ketonuria)’). A modified list of clinical discriminators for the use of prehospital providers that are less subjective, require no diagnostics and fall within the scope and training of prehospital providers has the potential for improving prehospital triage in systems like WCG EMS.

Participants also reported situations in which their clinical gestalt or level of concern for a patient was discordant with the final SATS acuity. BLS and ILS providers discussed using free text discriminators to request additional field resources, such as an ALS ambulance “upgrade”, in situations where they feared the patient could decompensate (e.g., patients with significant injuries). All types of providers suggested they create their own discriminator and then manually upgrade their assessed SATS to match their level of concern and destination facility choice. The use of SATS as a decision tool for destination facility will inherently have mismatches due to the conflict between the original hospital-oriented intent and prehospital application. Perhaps, allowing for EMS senior ALS or ILS provider discretion to upgrade the SATS acuity may better allow them to match patients with their prehospital-determined acuity.

### Special EMS considerations

Prehospital providers are faced with the challenges of being advocates for their patients, safeguarding health system resource utilization and applying their own clinical assessment. Not infrequently, these priorities come into conflict. Participants noted pressure from patients to modify their triage or destination due to social concerns and healthcare resource needs. While SATS or the EMS provider’s clinical judgement might dictate that a patient from a rural area be transported to a distant referral centre, some patients may lack the resources to return from such long distances and plead to be taken to a closer day hospital. Reconciling SATS destination decisions with patient preference requires clear guidance that respects patient autonomy while serving the overall system.

The potential for a change in clinical status of a patient during prolonged transportations also became apparent in focus groups. On the one hand, the fear of decompensation of their patient may drive providers to try to upgrade the SATS to request further resources (e.g. ALS providers) on scene or transport to a closer (under-resourced) facility, while improvements in a patient’s clinical status during transport (e.g. a hypoglycaemic patient that responds to glucose) may provoke a receiving facility to suggest the patient’s acuity no longer warrants that level of care and suggest they transport to another facility. This creates inefficiencies for the EMS system and frustrations among EMS providers. Integrating SATS into EMS operational policies, and aligning prehospital SATS with local destination guidelines, may help improve this issue.

### Limitations and generalizability

While this study was done with one EMS system and may not be generalizable to other EMS systems, the authors feel there are many lessons learned that apply to other EMS systems in limited resource settings that require a prehospital triage tool that has to meet multiple, often conflicting, demands. The challenges identified by participants regarding implementation and use of SATS are important for other EMS systems considering using SATS or another hospital based triage tool in the prehospital setting. Potential solutions or modifications such as modification of the discriminator list and separate or integrated protocols that address challenging sub-populations like trauma would likely apply to other EMS systems.

While the sample size and convenience sampling method inherently limit the data, we were able to recruit a diverse collection of providers with varying experience in rural vs urban setting, level of training, and duration of employment with WCG EMS. Despite having only three focus groups, we reached thematic saturation. The qualitative design and analysis was focused to assess the prehospital providers’ experience with SATS in their setting. As such, we did not investigate the perception of prehospital SATS from in-hospital personnel or dispatchers, both of which interact routinely with the study group as they use SATS.

## Conclusion

This study was the first to evaluate prehospital providers’ perspectives and experience with the use of the SATS. WCG EMS providers of all levels of training generally expressed positive attitudes regarding SATS, including objectivity of TEWS and benefits of having a common triage tool with hospital providers. However, respondents identified significant gaps and limitations to effectively using SATS in the prehospital setting, including poor applicability of many clinical discriminators, aligning prehospital providers’ perceived severity with SATS triage acuity, non-integration of SATS into destination guidelines, and challenges using SATS in rural areas or with long-distance transports. Together, these findings suggest that prehospital SATS use may be improved by on-going training, prehospital modifications of the discriminator list and better integration of SATS with local destination guidelines.

## Supplementary Information

**Additional file 1 **: Interview Guide**.** Assessing use of South African Triage Scale (SATS) by Western Cape EMS

## Data Availability

The datasets used and/or analysed during the current study are not publicly available but are available from the corresponding author on reasonable request.

## Abbreviations

ALS: Advanced Life Support
BLS: Basic Life Support
EMS: Emergency Medical Services
ILS: Intermediate Life Support
SATS: South African Triage Scale
TEWS: Triage Early Warning Score
WCG: Western Cape Government
