# Supplementary material for: Using the South African Triage Scale for prehospital triage: a qualitative study
Source: BMC Emerg Med. 2021 Oct 30;21:125. doi: 10.1186/s12873-021-00522-3 (PMC8556887; doi:10.1186/s12873-021-00522-3)
Supplement: Supplementary file 1 — Additional file 1 : Interview Guide. Assessing use of South African Triage Scale (SATS) by Western Cape EMS [file 12873_2021_522_MOESM1_ESM.docx]

**Interview Guide**

**TITLE:** Assessing use of South African Triage Scale (SATS) by Western Cape EMS

**FACILITATORS:** ____________________________________________________________

**DATE:** ______________ **TIME:** __________

**LOCATION**:___________________________

**COHORT:** EMS Medics [ ]ALS [ ]ILS [ ] BLS Number of participants: __________

**Introductory Script:**

- Thank you for taking the time to discuss with me your opinions about TEWS and SATS in the prehospital environment.
- I’m (facilitator name), and I will be facilitating the discussion
- This is (other facilitators’s name(s)) and he/she will be taking notes and observing.
- I am a doing research through the University of Cape Town and the University of Colorado to better understand how to improve triage in the prehospital environment.
- There are no ‘right’ or ‘wrong’ answers to any of the things we talk about today.
- This group discussion is designed to learn more about your knowledge, experiences, feelings and opinions.
- You can respond to each other, this is meant to be a discussion
- I’m interested in talking about this with you because I would like to improve care provided to patients and training of EMS providers.
- The discussion will last about one hour.
- All of your responses will be kept private and confidential.
- The discussion will be audio recorded, so I don’t have to take so many notes.
- However, no names or identifying information will be kept on this recording.

**Before we begin, let me mention a few things about how we usually conduct these groups:**

- My role is to ask the questions we have for the group and to encourage everyone to participate.
- I won’t be doing much talking, but may ask you to explain more or to give an example. Also, it’s my job to see that everyone has a chance to voice their opinions, as well as to keep us moving along so that we have time to discuss all of the questions.
- So, at times, it might seem as though I am cutting you off, and this is not meant to be rude, but rather to make sure that we have time to have a complete discussion of each question.
- THERE ARE NO RIGHT OR WRONG ANSWERS!!!
- Each person’s experiences and opinions are valid, and we want to hear a wide range of opinions on the questions we’ll be asking.
- Please speak up, whether you agree or disagree, and let us know what you think.

- Sometimes participants bring up sensitive issues during these discussions
- Anything of a personal nature that is mentioned in this room will NOT be repeated to others outside of this discussion group.
- Can I see a nod from everyone showing me that you agree with this confidentiality ground rule?
- Facilitator: If anyone is not willing to give their consent to confidentiality, they may be excused from the group.
- We are using 2 digital recorders to record our discussion.
- We want to capture everything that all of you say, and we simply can’t write fast enough to get it all down.
- We’ll be using only first names in the group discussion, and when we put together the results from all the groups, we won’t include any names at all.
- It is very important that we speak one at a time, so that the recorder picks up everything that is said.
- Is everyone OK with being recorded?
- Can everyone say hello so that we can recognize your voice on the recording
- I’m also going to pass out note cards to each of you.
- On these notecards please write your age, gender, years of field experience in Western Cape EMS and current level of training.
- I will collect these at the end of the session.
- If there is any feedback that you would prefer not to share out loud, that you prefer not to be recorded, or that you didn’t have a chance to say, please write it down on these cards and then give them to us after the focus group is over or talk to me after the focus group is over.
- Anything that you say during the group and decide that you don’t want to be included on the recording will be deleted from the recording.
- Just let us know after the session if there is anything that you said that you would like deleted.

- We plan to be finished with our discussion by (time). Are there questions about any of this?
- Please be sure to shut off any phones/pagers you have with you tonight.
- Lastly, we need you to understand that the focus group is not an educational session where we give advice.
- Let’s get started.”

Interview guide for focused group discussion:

1. **Tell me about how you were originally trained or taught about calculating and using TEWS and SATS** and if you ever received any additional training
2. Tell me about feedback you get on your use of TEWS and SATS.
3. Tell me a little more about what you know about the South African Triage Scale (SATS)

*Anything else you would like to bring up about SATS?*

1. Tell me about some cases that were easy or convenient to calculate TEWS and SATS.
2. **Tell me about some cases that were difficult to use TEWS and SATS?**
   1. What about those cases made them particularly challenging?
3. **What are some other challenges to using SATS in the prehospital setting?**
   1. Calculating TEWS…
   2. Using discriminators…
   3. Final SATS colour…
4. **Do you think calculating TEWS and SATS is helpful to patients? Why?**
5. **What do you think are the benefits of using TEWS and SATS as a medic?**
6. Do you think that SATS triages patients accurately? Why or why not?
7. We have found that Western Cape EMS medics incorrectly calculate SATS about 2/3’rds of the time. Do you have any ideas why that might be?
   1. Documentation- if yes, explain more
   2. Too much time
8. Where do you think the challenges lie, do you think SATS is an appropriate tool? Why or why not?
9. Can you tell me a few ways we can make it easier for you to use TEWS & SATS more often and accurately? Are there any ways you think you could use the information from SATS better?
10. If you could design a triage tool, what parts would you include? What parts would you not include?
11. What parts do you think are the most important for patients? Most important for medics?

Is there anything else you would like to discuss? Anything you wished we had asked about?
